# Machine-Specific Magnetic Resonance Imaging Quality Control Procedures for Stereotactic Radiosurgery Treatment Planning

**DOI:** 10.7759/cureus.1957

**Published:** 2017-12-18

**Authors:** Ali Fatemi, Somayeh Taghizadeh, Claus Chunli Yang, Madhava R. Kanakamedala, Bart Morris, Srinivasan Vijayakumar

**Affiliations:** 1 Radiation Oncology/radiology, University of Mississippi Medical Center; 2 Radiation Oncology, University of Mississippi Medical Center

**Keywords:** mri, qc, srs treatment planning, mri guided radiotherapy, mri qa

## Abstract

Purpose

Magnetic resonance (MR) images are necessary for accurate contouring of intracranial targets, determination of gross target volume and evaluation of organs at risk during stereotactic radiosurgery (SRS) treatment planning procedures. Many centers use magnetic resonance imaging (MRI) simulators or regular diagnostic MRI machines for SRS treatment planning; while both types of machine require two stages of quality control (QC), both machine- and patient-specific, before use for SRS, no accepted guidelines for such QC currently exist. This article describes appropriate machine-specific QC procedures for SRS applications.

Methods and materials

We describe the adaptation of American College of Radiology (ACR)-recommended QC tests using an ACR MRI phantom for SRS treatment planning. In addition, commercial Quasar MRID^3D^ and Quasar GRID^3D^ phantoms were used to evaluate the effects of static magnetic field (B_0_) inhomogeneity, gradient nonlinearity, and a Leksell G frame (SRS frame) and its accessories on geometrical distortion in MR images.

Results

QC procedures found in-plane distortions (Maximum = 3.5 mm, Mean = 0.91 mm, Standard deviation = 0.67 mm, >2.5 mm (%) = 2) in X-direction (Maximum = 2.51 mm, Mean = 0.52 mm, Standard deviation = 0.39 mm, > 2.5 mm (%) = 0) and in Y-direction (Maximum = 13. 1 mm , Mean = 2.38 mm, Standard deviation = 2.45 mm, > 2.5 mm (%) = 34) in Z-direction and < 1 mm distortion at a head-sized region of interest. MR images acquired using a Leksell G frame and localization devices showed a mean absolute deviation of 2.3 mm from isocenter. The results of modified ACR tests were all within recommended limits, and baseline measurements have been defined for regular weekly QC tests.

Conclusions

With appropriate QC procedures in place, it is possible to routinely obtain clinically useful MR images suitable for SRS treatment planning purposes. MRI examination for SRS planning can benefit from the improved localization and planning possible with the superior image quality and soft tissue contrast achieved under optimal conditions.

## Introduction

Using magnetic resonance (MR) images for stereotactic radiosurgery (SRS) treatment planning requires careful consideration of a number of factors [1], including choice of the correct magnetic resonance imaging (MRI) pulse sequences (3D, no slice gap, less geometrical distortion, high signal-to-noise ratio, and isotropic spatial resolution), immobilization devices (MRI-compatible SRS frame), customized radiofrequency (RF) coils (proper sensitivity, low RF deposition, and consequently less contour deformation) such as a single channel send-and-receive RF head coil (Rx/Tx RF head coil), and most importantly, confirmation that the MRI images acquired possess high geometrical accuracy and stability.

Existing MR quality control (QC) procedures [2-6] are inadequate for assessing MRI scanners for SRS treatment-planning purposes, primarily because existing tests have been developed for machines used in general diagnostic radiology. There, the goal is to maintain image quality rather than spatial fidelity and signal intensity [7]. Several well-established references from the American College of Radiology (ACR) [2,3] and the American Association of Physicists in Medicine (AAPM) [4-6] provide guidance regarding QC procedures for MRI scanners used in diagnostic radiology, but no guidance documents currently describe the unique QC factors that must be considered when using MRI scanners in SRS treatment planning [7-9]. However, existing quality control tests can be modified to provide the necessary information for a given SRS-planning application by testing over appropriate volumes and using SRS-specific MR imaging parameters.

Development of our QC process started with an evaluation of gross machine factors, including main static magnetic field (B_0_) inhomogeneity and gradient non-linearity over a large field of view using the scanner body coil, then narrowed to study the effects on the geometrical stability of MR images due to use of an MRI-compatible SRS frame and its localizer using a Tx/Rx RF head coil [10]. Finally, adapted ACR tests were performed to evaluate the image contrast, spatial resolution, gradient stability for accurate slice selection and thickness, RF coil sensitivity, and acquisition of artifact-free MR images. Testing these factors ensures that acquired images possess the quality and resolution required for precision SRS treatment planning, accurately identifying disease extent and proximity relative to adjacent organs at risk (OAR) [1].

We are establishing a quality assurance (QA) program to continuously and systematically evaluate MRI scanner performance, safety and stability for SRS treatment planning. Our goal in this article is to describe our QC tests and strategy in establishing a QA program for MRI-guided SRS treatment planning. This paper focuses narrowly on MRI machine-specific aspects of the QC procedure and leaves patient-specific QC tests, including patient-specific geometrical distortion evaluation, correction methods, customized RF coils, patient comfort, MRI safety, and MRI pulse sequence optimization, for future reports.

## Materials and methods

We recently installed a Leksell Gamma Knife® Icon™ SRS treatment unit (Elekta AB, Stockholm, Sweden) and a MAGNETOM Aera 1.5 T (Siemens Healthcare, Germany) Radiotherapy Edition MRI machine at our institute. The SRS committee consists of three physicists, a radiologist, a radiation oncologist, and a neurosurgeon, who work together to develop guidelines for MRI-guided SRS treatment planning. MR images are used to assess cases of brain metastasis, pituitary/parasellar lesions, acoustic neuroma, trigeminal neuralgia and arteriovenous malformation (AVM).

The MRI SRS QC procedure has been developed based on factors including imaging site, MRI pulse sequence(s), adapted or standard RF coils, and any immobilization devices required. SRS patients are scanned on a regular diagnostic MRI table, using a Leksell G frame with immobilization and localization devices, or frameless, as appropriate. In our institute, we use a Tx/Rx CP head coil (Siemens Healthcare, Germany) to fit the Leksell G frame, plus an MRI indicator box with an adaptor to the coil, and are still able to keep the specific absorption rate (SAR) under 3 W/kg. The downside of using such an RF coil is a less-than-ideal signal-to-noise ratio (SNR) and long scanning time; thus, we use a regular 20-channel RF head coil for frameless cases. MRI pulse sequences have been evaluated by the SRS committee based on disease site and treatment planning criteria detailed in Table 1.

**Table 1:** Approved MRI pulse sequences for SRS treatment planning. CISS: Three-dimensional (3D) constructive interference in steady state; MPRAGE: Magnetization-prepared 180-degree radio-frequency pulses and rapid gradient-echo; MRI: Magnetic resonance imaging; SRS: Stereotactic radiosurgery; TE: Time of Echo; TR: Time of Repetition.

Sequence/contrast	Parameters	Disease
Axial T1-weighted MPRAGE	1 x 1 x 1 mm^3^, TR/TE = 2200/2.91 ms, 300 Hz/pixel	Brain metastasis, pituitary/parasellar lesions, acoustic neuroma/schwannoma, trigeminal neuralgia, arteriovenous malformation
Axial T2-weighted Space	0.9 x 0.9 x 1 mm^3^, TR/TE = 1400/184 ms, 345 Hz/pixel	Pituitary/parasellar lesions, acoustic neuroma/schwannoma, arteriovenous malformation
Axial T2 CISS	0.9 x 0.9 x 1 mm^3^, TR/TE = 5.48/2.38 ms, 340 Hz/pixel	Pituitary/parasellar lesions, acoustic neuroma/schwannoma, trigeminal neuralgia

We summarize the commissioning and QC tests (test, frequency, and machine tolerance) in Table 2 and Table 3.

**Table 2:** Quality control tests and frequencies for magnetic resonance imaging-guided stereotactic radiosurgery. ACR: American College of Radiology; B_n_: Magnetic field; MRI: Magnetic resonance imaging; PMU: Phasor measurement unit; QA: Quality assurance; RF: Radiofrequency; Rx: Receive; SRS: Stereotactic radiosurgery; Tx: Transmit.

Daily QA (MRI technologists) using ACR phantom	Monthly QA (Therapy physicist/MRI physicist) using MRID^3D^, GRID^3D^ and ACR phantoms	Annual QA (MRI physicist) using MRID^3D^, GRID^3D^ and ACR phantoms
Inspect bore for loose metal (bobby pins, earrings, etc.)	Patient safety (monitors, intercom, panic ball, emergency buttons, and signage)	20-channels RF coil integrity check
Tx/Rx and 20-channel RF coil SRS check using uniform phantom	Patient comfort (bore light and fan)	B_0_ constancy
Patient safety (intercom, panic ball, detector)	Percent signal ghosting	B_1+_ constancy
Geometry accuracy and B_0_ check using ACR phantom	Percent image uniformity	Gradient linearity constancy
	High/low contrast accuracy	Slice thickness accuracy
Weekly QA (MRI technologists) Using ACR phantom	Coach position accuracy	Slice position accuracy
Transmitter gain constancy	Image artifact	Geometrical accuracy
Center frequency constancy	Geometrical accuracy (large field of view)	Rx/Tx RF head coil check
20-channel RF head coil SNR	Geometrical accuracy (small field of view) with and without frame	20-channel RF head coil check
Rx/Tx RF head coil SNR		Dynamic field map
Slice thickness accuracy		Eddy current compensation
Slice position accuracy		Gradient delay
Geometric accuracy and B_0_ check using ACR phantom		Gradient sensitivity
		Body coil image brightness
		Magnet shim
		Rx gain calibration
		Body coil tuning
		Spike
		PMU transmit
		Rx stability
		Tx stability

**Table 3:** Summary of magnetic resonance imaging quality control tests for stereotactic radiosurgery treatment planning. DSV: Diameter of spherical volume; MRI: Magnetic resonance imaging; PIU: Percent integral uniformity; PMU: Phasor measurement unit; PSG: Percentage signal ghosting; RF: Radio frequency; Rx/Tx: Receive/transmit; SNR: Signal-to-noise ratio.

Test	MRI machine tolerance
MRI geometrical distortion	
Evaluate distortion vector, combined effect (B_0_ inhomogeneity and gradient nonlinearity) over big field of view (37 cm)	<1 mm over 20 cm DSV and <2 mm over 37 cm DSV
Evaluate B_0_ inhomogeneity over large field of view (37 cm)	2 ppm
Evaluate the geometrical distortion vector with stereotactic frame (small field of view, 20 cm)	<1 mm
Adapted ACR QC tests	
Setup and table position accuracy	<1 mm
Center frequency	Pass/Fail
Signal ghosting	≤2.5%
Transmitter gain or attenuation	Pass/Fail
High contrast spatial resolution	Row and column resolution ≤ 1 mm
Low contrast detectability	Nine rows total for up to 1.5 T
Magnetic field homogeneity	Action limit ± 2 ppm
Artifact evaluation	Pass/Fail
Magnetic field homogeneity	Action limit ± 2 ppm
Geometrical accuracy	Within < 1.5 mm of actual length
Visual checklist	Pass/Fail
Slice position accuracy	Difference from actual position ≤ 3 mm
Slice thickness accuracy	Action limit is 5 ± 0.7 mm
20-channel RF head coil evaluation	Signal-to-noise ratio; PIU ≥ 87.5% (<3 T), PSG ≤ 2.5%
Rx/Tx RF head coil evaluation	Signal-to-noise ratio; PIU ≥ 87.5% (<3T), PSG ≤ 2.5%
Rx/Tx RF head coil check	Pass/Fail
20-channel RF head coil check	Pass/Fail
Dynamic field map	Pass/Fail
Eddy current compensation	Pass/Fail
Gradient delay	Pass/Fail
Gradient sensitivity	Pass/Fail
Body coil image brightness	Pass/Fail
Magnet shim	Pass/Fail
Rx gain calibration	Pass/Fail
Body coil tuning	Pass/Fail
Spike	Pass/Fail
PMU transmit	Pass/Fail
Rx stability	Pass/Fail
Tx stability	Pass/Fail
Assessment of MRI safety program	Pass/Fail

Evaluation of geometrical distortion over a large field of view

**Figure 1:**
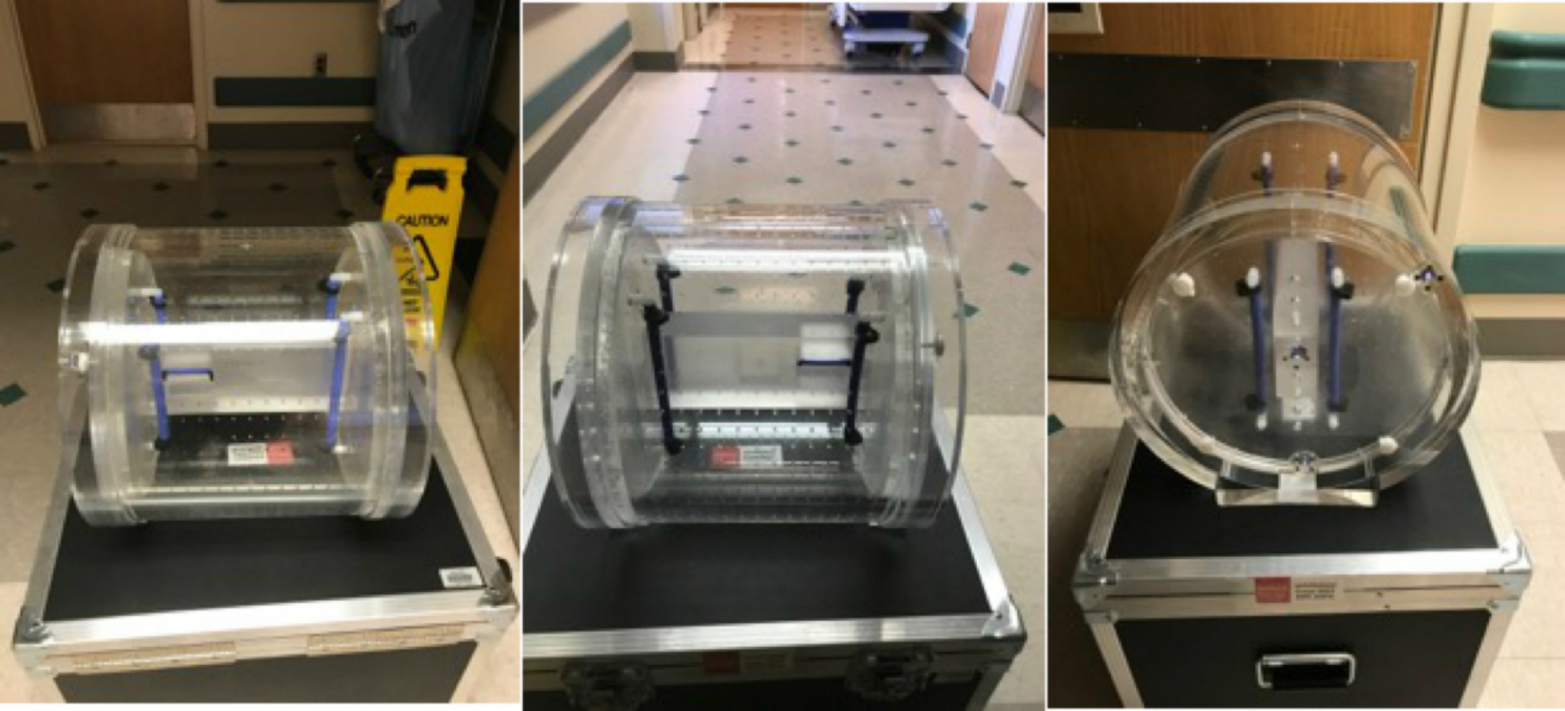
Quasar MRID3D geometrical distortion phantom (Modus Medical).

We used a QUASAR™ MRID^3D^ geometrical distortion phantom (Figure 1) (Modus Medical, Canada) to measure B_0_ inhomogeneity and gradient non-linearity using a reverse gradient technique over a 37 cm x 32 cm (W x L) phantom area. The phantom was scanned with a 3D VIBE T1-weighted sequence: 1 mm^3^ isotropic voxels, NEX of two, TE of 4 ms, TR of 9 ms, a flip angle ~10°, and a bandwidth of 120 Hz/pixel. QUASAR™ MRID^3D^ comes with easy-to-use image analysis software for calculation of the phantom boundary distortion vector field, volumetric 3D distortion vector field, and B_0_ distortion vs gradient distortion, using 3D spherical harmonic analysis.

**Evaluation of the effect of an SRS frame and localizer on geometrical stability of MR images**

**Figure 2:**
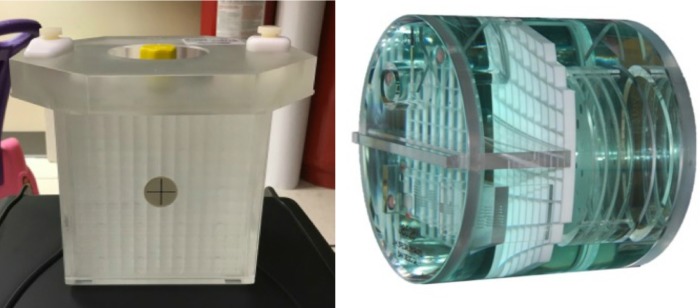
Phantoms used in the study. Left: Quasar GRID^3D^ image distortion phantom (Modus Medical, Canada); Right: Standard American College of Radiology magnetic resonance imaging phantom.

We used a QUASAR™ GRID^3D^ Image Distortion Phantom and analysis system (Modus Medical, Canada) to evaluate MR image distortion due to the introduction of an SRS frame and localizers. The system is comprised of a phantom and analysis software which work together to produce a 3D map of spatial distortion with submillimeter accuracy throughout a volume of interest. The phantom (Figure 2, left) is an acrylic cube containing a 1-cm 3D grid of channels filled with copper sulfate solution. The region of interest is a 14 x 13 x 11 cm^3^ volume containing 2002 vertex locations, the positions of which are known to within 0.1 mm. The phantom accurately and reproducibly mounts securely to the SRS Leksell Frame G at a known position. It fits within both the Leksell® MR Indicator and Leksell® CT Indicator. We scanned our phantom using a 3D MPRAGE pulse sequence: T1-weighted, 1 x 1 x 1 mm^3^, TR/TE of 2200/3.74 ms, and 350 Hz/pixel. The MPRAGE is the only MRI pulse sequence currently being used for treatment planning, and the rest of the sequences will be registered rigidly.

ACR MRI tests adapted for SRS treatment planning

We used a standard MRI ACR phantom (Figure 2, right) to evaluate the rest of our adapted QC MRI tests. The ACR phantom has been scanned based on MRI pulse sequences and parameters summarized in Table 4. All QC tests with their tolerances are summarized in Table 3.

**Table 4:** MRI pulse sequences and parameters for adapted magnetic resonance imaging ACR quality control tests. ACR: American College of Radiology; BW: Bandwidth; FOV: Field of view; MRI: Magnetic resonance imaging; NEX: Number of excitation; TE: Time of Echo; TR: Time of Repetition. *Acquired three separate series, each consisting of a single image through the center of phantom with minimum and highest bandwidths.

Protocol	Phantom used	TR (ms)	TE (ms)	FOV (mm)	# Slices	Slice thickness (mm)	GAP		NEX	Matrix	BW
ACR T1 Localizer	ACR	200	20	25	1	20	N/A		1	256 x 256	Routine (15.6 kHz)
ACR T1 Axial	ACR	500	20	25	11	5	5		1	256 x 256	Routine (15.6 kHz)
ACR T2 Axial	ACR	2000	20/80	25	11	5	5		1	256 x 256	Routine (15.6 kHz)
Site T1 Axial	ACR	Site protocol	Site protocol	25	11	5	5		Site protocol	256 x 256	Site protocol BW
Site T2 Axial	ACR	Site protocol	Site protocol	25	11	5	5		Site protocol	256 x 256	Site protocol BW
Low BW Axial *	ACR	500	20	25	1	5	N/A		1	256 x 256	Minimum BW @ 256 x 256 matrix
Low BW Coronal *	ACR	500	20	25	1	5	N/A		1	256 x 256	Minimum BW @ 256 x 256 matrix
Low BW Sagittal*	ACR	500	20	25	1	5		N/A	1	256 x 256	Minimum BW @ 256 x 256 matrix
High BW Axial*	ACR	500	20	25	1	5		N/A	1	256 x 256	Highest BW @ 256 x 256 matrix
High BW Coronal*	ACR	500	20	25	1	5		N/A	1	256 x 256	Highest BW @ 256 x 256 matrix
High BW Sagittal*	ACR	500	20	25	1	5		N/A	1	256 x 256	Highest BW @ 256 x 256 matrix

Evaluating MRI safety

As part of our acceptance tests, we used a gauss meter to carefully map and post the five-gauss line with proper signage. We monitor all patients through both a questionnaire and in-person consultation to ensure that any person with a cardiac pacemaker or neurosimulators does not cross the five-G line. Our MRI room is also equipped with a Ferroguard® wall-mounted system deployed in an entryway mode on both sides of the doorway. This system provides real-time monitoring of the local ferromagnetic environment with an audible alert system. We also check the patient/console intercom system, table-top button (magnet housing and console), emergency stop buttons, emergency rundown unit, and door switches on a regular basis.

Establishing the MRI quality assurance program

We summarize our proposed QC tests and their frequencies in Table 2. What follows is a formulaic approach to monitor B_0_ inhomogeneity and geometrical distortion with weekly and daily QC tests using an ACR phantom. We found that incorporating these tests with the recommended weekly ACR tests run by a technologist and using only an MRI ACR phantom makes the process faster and more efficient in our busy clinic.

First, we defined our reference B_0_ inhomogeneity and geometrical distortion during monthly and commissioning processes using Quasar MRID^3D^. The MRI image geometrical distortion and machine B_0_ inhomogeneity were defined over a 37 cm x 32 cm (W x L) area in three dimensions (D_x_, D_y_, D_z_) and absolute value from MRI isocenter. Next, we scanned the regular ACR MRI phantom and defined B_0_ inhomogeneity using a bandwidth difference technique, and defined geometrical distortion using sagittal slices one and five for all three dimensions. We used geometrical distortion measurements for slice 5 (D_x_, D_y_) and the sagittal plane (D_z_) for baseline calculation, assuming that slice five is at or very close to the MRI isocenter. Finally, the average baseline was defined based on equations 1 and 2 for the same slices and diameter on both MRID^3D^ and ACR phantoms. The baseline measure is used for weekly checks, and we define our tolerance as 2% changes, and action level as a measured 4% difference.

Equation 1: Base B_0_ = ACR_B0_ − MRID^3D^_B0_

Base B_0_ is an averaged B_0_ inhomogeneity at the same slice at MRID^3D^ and ACR phantoms; MRID^3D^_B0_ is the measured average B_0_ inhomogeneity in ppm; and ACR_B0_ is averaged B_0_ inhomogeneity (ppm) using the bandwidth difference technique, and

Equation 2: Base_geometrical distortion_ = ACR_geometrical distortion_ – MRID^3D^_geometrical distortion_

Where Base_geometrical distortion_ is the geometrical distortion at the slice and orientation at MRID3D and ACR phantoms; ACR_geometrical distortion_ is the measured geometrical distortion at slice five and sagittal plane on all three directions (D_x_, D_y_, D_z_); and MRID^3D^_geometrical distortion_ is the measured geometrical distortion at the same ACR slice and orientation.

## Results

The geometrical distortion over a 37 cm x 32 cm (W x L) area was evaluated in all three dimensions (D_x_, D_y_, D_z_), absolute distance from MRI isocenter. Table 5 contains summary statistics; the maximum distortion in the x and y plane (axial plane) was 3.5 mm and 2.5 mm at the boundaries.

**Table 5:** Summary statistics for MRID3D geometrical distortion measurements. STD: Standard deviation.

	Mean (mm)	STD (mm)	Max (mm)	>2.5 mm (%)
dx	0.91	0.67	3.5	2
dy	0.52	0.39	2.51	0
dz	2.38	2.45	13.1	34
dr	2.79	2.36	13.19	40

**Figure 3:**
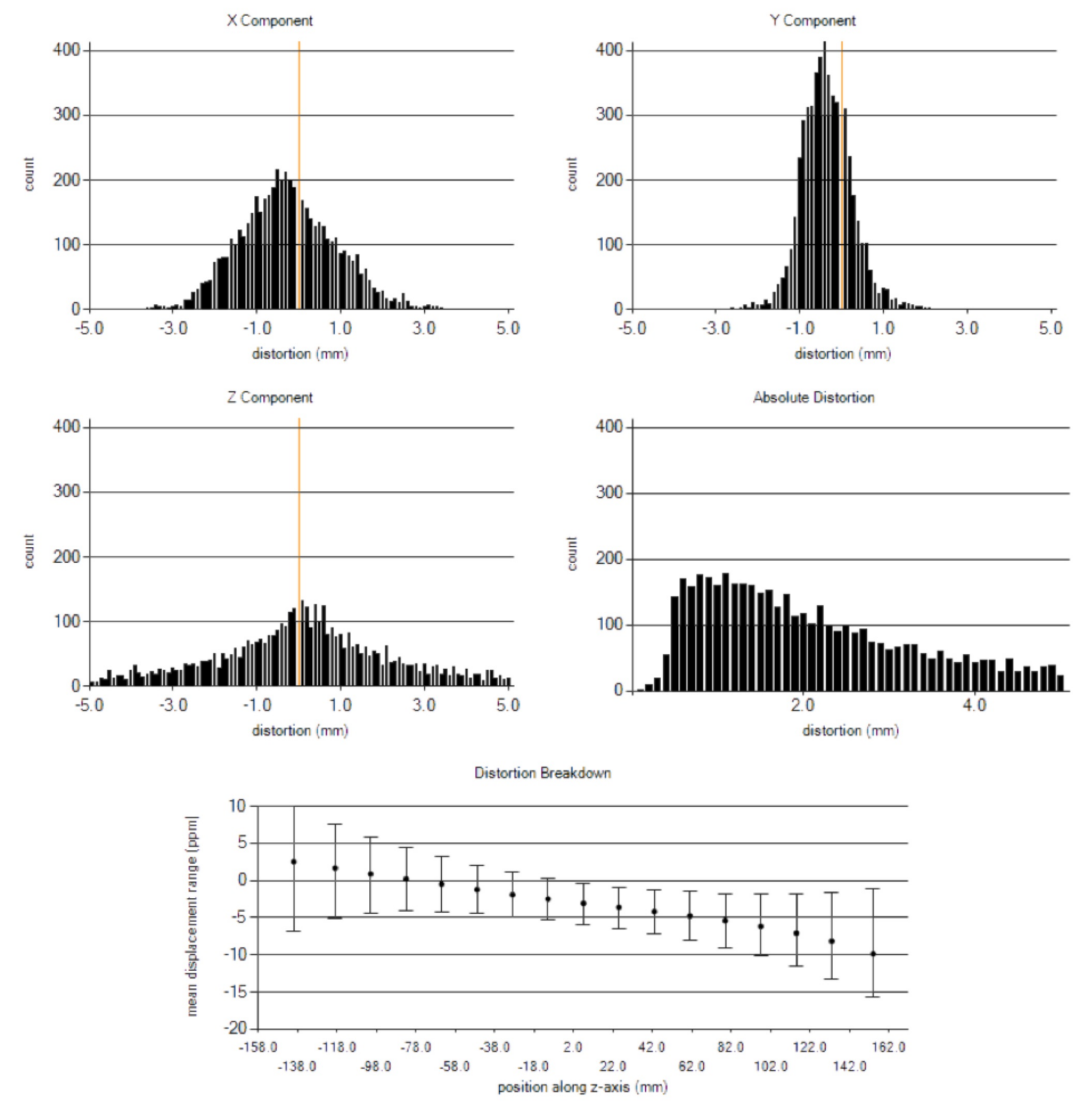
Static magnetic field (B0) inhomogeneity and distortion measurement results using an MRID3D phantom.

The detailed measurements along all three coordinates and their absolute values with respect to MRI isocenter are shown in Figure 3. The B_0_ inhomogeneity along the z-direction was measured separately using an inverse gradient technique, and those data are also shown in Figure 3.

We used a QUASAR™ GRID^3D^ Image Distortion Phantom and analysis system (Modus Medical, Canada) to evaluate image distortion in MR images due to the presence of an SRS frame and localizers. The data in the axial plane of the MRI images showed a maximum of 0.5 mm in the x-direction, 1.5 mm in the y-direction; in the z-direction, the maximum of 2.6 mm was observed at the phantom boundary (11 cm from MRI isocenter). The results showed a mean absolute deviation of 2.3 mm from isocenter. We defined our ACR phantom weekly B_0_ inhomogeneity and geometrical distortion baselines: the reference B_0_ inhomogeneity using MRID^3D^ phantom was evaluated on all three axes (axial: 1.89 ppm, coronal: 0.135 ppm and sagittal: 0.068 ppm) as well as on average (0.699 ppm), all well-defined within our limits of ±2 ppm. The *Base_B0_* inhomogeneity based on Equation 1 was defined as 0.03 ppm. The Base_geometrical_ _distortion_ was defined for our MRI ACR phantom at slice 5 as 0.6 mm in the x-direction, 0.8 mm in the y-direction, and 0.5 mm in the sagittal plane.

All adopted ACR measures were within our defined tolerance, as summarized in Table 6. Specifically, the 20-channel and Rx/Tx RF head coils have been tested thoroughly for SNR, percent integral uniformity (PIU) and percentage signal ghosting (PSG), and the results were found to fall within our accepted limits.

**Table 6:** Magnetic resonance imaging stereotactic radiosurgery planning quality control results. BW: Bandwidth; MRI: Magnetic resonance imaging; PMU: Phasor measurement unit; RF: Radiofrequency; Rx/Tx: Receiver/transmitter; PIU: Percent image uniformity; SNR: Signal-to-noise ratio.

MRI equipment evaluation summary			
	Setup and table position accuracy		Pass
	Center frequency		Pass
	Transmitter gain or attenuation		Pass
	Geometric accuracy measurements*		Pass
	High-contrast spatial resolution*		Pass
	Low-contrast detectability*		Pass
	Artifact evaluation		Pass
	Visual checklist		Pass
	Magnetic field homogeneity		Pass
		Method of Testing	BW diff
	Slice-position accuracy*		Pass
	Slice-thickness accuracy*		Pass
	Radiofrequency coil checks (20-channel RF head coil)		
		a. SNR	Pass
		b. Volume coil percent image uniformity	Pass
		c. Percent signal ghosting	Pass
	Radiofrequency coil checks (Rx/Tx RF head coil)		
		a. SNR	Pass
		b. Volume coil PIU	Pass
		c. Percent signal ghosting	Pass
	Rx/Tx RF head coil check		Pass
	20-channel RF head coil check		Pass
	Dynamic field map		Pass
	Eddy current compensation		Pass
	Gradient delay		Pass
	Gradient sensitivity		Pass
	Body coil image brightness		Pass
	Magnet shim		Pass
23	RX gain calibration		Pass
24	Body coil tuning		Pass
25	Spike		Pass
26	PMU transmit		Pass
27	Rx stability		Pass
28	Tx stability		Pass

## Discussion

The methodology discussed herein describes practical strategies we have implemented through lessons learned performing clinical MRI QA and SRS treatment planning [11-14]. We focus on discussion of major issues encountered during our QC procedures.

The MRI machine specifications which have the highest potential to affect SRS treatment planning are B_0_ and B_1_ inhomogeneity and gradient non-linearity, which affect the geometrical accuracy and intensity uniformity of MRI images. Use of single-channel Rx/Tx RF head coils, a Leksell G frame (SRS frame) and accessories for SRS treatment planning only exacerbates these issues. Using an MRID^3D^ phantom over a 37 cm x 32 cm (W x L) area gives enough information about MRI image distortion due to B_0_ inhomogeneity and gradient non-linearity to allow acquired images to be used for SRS treatment planning. As we expected, geometrical distortion is within 1 mm accuracy in the axial plane (x- and y-directions), and 2 mm along the z-direction, 10 cm from isocenter (almost head size), but it worsens to the order of 5 mm at the boundaries (16 cm away from isocenter).

Immobilization devices constructed from materials optimized for radiation therapy may not necessarily be optimal for MRI (e.g., carbon fiber) [15-17]. In our experience, it is no longer sufficient for immobilization device materials (Leksell G frame, screws, adaptor and MRI localizer) to be simply MRI-compatible; these materials and devices should be MRI-optimal. Poor material choices can contribute to magnetic susceptibility-induced geometric distortions. Our phantom results specifically on 3D axial T2 SPACE (Sampling Perfection with Application optimized Contrasts using different flip angle Evolution) and axial T2 CISS (Three-dimensional constructive interference in steady state) sequences show artifacts even after pulse sequence optimization and use of different orientations. It is essential that some MRI sequences reviewed by the SRS team be repeated using a different sequence, such as 2D axial T2 or T1-weighted Turbo Spin Echo (TSE). However, our results from Quasar™ GRID^3D^ shows that images acceptable for treatment planning can be obtained with the use of Laksell frame and localization devices by using the right MRI pulse sequence and a Tx/Rx RF head coil.

Our proposed SRS MRI QA program has been reviewed and approved by our QA committee, and peer reviewed at every step by SRS committee members. Our aim is to minimize the scanning time and maximize efficiency. One major change proposed was the use of MRI ACR phantom for weekly geometrical accuracy checks rather than the MRID^3D^. This streamlines the process and the technologist can incorporate these results into the regular weekly checks.

Our results indicated that gradient nonlinearity-induced geometric distortions can be severe and must be corrected using 3D distortion correction prior to using MR images for SRS treatment planning. However, even with 3D distortion correction, residual distortions can persist for large FOV prescriptions. One compounding factor is that some MRI scanners permit acquisition of image volumes positioned off-center from isocenter in the superior/inferior direction. This approach increases the likelihood of scanning in regions of nonlinear gradients and, therefore, increases the likelihood of residual distortions. At a minimum, the magnitude of these residual distortions should be characterized as a function of radial distance from isocenter for each scanner. Ideally, the residual distortions would be corrected.

High MRI image intensity uniformity is critical in SRS treatment planning. Phased-array RF coils require correction for differences in the sensitivity profiles of each coil element during reconstruction to optimize image uniformity. These revisions, often based on a quick prescan image, become increasingly important when flexible phased-array RF coils, wrapped around the patient in various positions, are utilized. Our results indicate that by using prescan normalization and post-processing corrections the MRI images collected are within preset limits and SNR, PIU and PSG tests serve as good indications for variation.

The participation of the dedicated SRS team, including the medical physicist, radiation oncologist, and neurosurgeon in the quantification, protocol modification and development of quality assurance procedures, as well as verification of MRI data used for SRS planning, is critical. Moreover, the scanner selection considerations, specifications, chosen MRI pulse sequences, and post-processing packages are critical in having a successful program of MRI-guided SRS treatment planning.

## Conclusions

In conclusion, we describe an MRI machine QC procedure to maintain clinically acceptable MR image acquisition for SRS treatment planning purposes. MRI examinations for SRS planning can benefit from the improved localization and planning possible with the superior image quality and soft tissue contrast achieved with appropriate MRI QA. We recommend convening a team of experts who meet periodically to review cases, discuss new MRI pulse sequences and technology, including newly available post-processing software packages, and who can develop a custom QA program for the facility. We firmly believe this type of dialog opens opportunities for advance use of MRI images in SRS treatment planning, especially in a new era of MRI-guided radiotherapy available in commercial machines.
